# Yoga for Persistent Fatigue in Breast Cancer Survivors: Results of a Pilot Study

**DOI:** 10.1155/2011/623168

**Published:** 2011-01-13

**Authors:** Julienne E. Bower, Deborah Garet, Beth Sternlieb

**Affiliations:** ^1^Department of Psychology, UCLA, Los Angeles, CA 90095, USA; ^2^Department of Psychiatry and Biobehavioral Sciences, Geffen School of Medicine, UCLA, Los Angeles, CA 90095, USA; ^3^Cousins Center for Psychoneuroimmunology, Semel Institute, UCLA, Los Angeles, CA 90095, USA; ^4^Division of Cancer Prevention and Control Research, Jonsson Comprehensive Cancer Center, UCLA, Los Angeles, CA 90095, USA; ^5^Pediatric Pain Program, Mattel Children's Hospital, UCLA, Los Angeles, CA 90095, USA

## Abstract

Approximately one-third of breast cancer survivors experiences persistent fatigue for months or years after successful treatment completion. There is a lack of evidence-based treatments for cancer-related fatigue, particularly among cancer survivors. This single-arm pilot study evaluated the feasibility and preliminary efficacy of a yoga intervention for fatigued breast cancer survivors based on the Iyengar tradition. Iyengar yoga prescribes specific poses for individuals with specific medical problems and conditions; this trial emphasized postures believed to be effective for reducing fatigue among breast cancer survivors, including inversions and backbends performed with the support of props. Twelve women were enrolled in the trial, and 11 completed the full 12-week course of treatment. There was a significant improvement in fatigue scores from pre- to post-intervention that was maintained at the 3-month post-intervention followup. Significant improvements were also observed in measures of physical function, depressed mood, and quality of life. These results support the acceptability of this intervention and suggest that it may have beneficial effects on persistent post-treatment fatigue. However, results require replication in a larger randomized controlled trial.

## 1. Introduction

Breast cancer is the most common cancer diagnosed in females, with over 252,150 new cases expected in the United States in 2008 [1]. With advances in detection and treatment, the number of women who survive breast cancer has increased significantly in recent years. Five-year survival rates for localized breast cancer have climbed to 98%, resulting in an estimated 2.6 million North American women living in the aftermath of breast cancer [1]. As survival times increase, addressing the impact of breast cancer and its treatment on long-term outcomes have become increasingly important [2]. In particular, better understanding and management of cancer-related symptoms is critical for reducing suffering in cancer survivors, as highlighted by a recent State of the Science conference sponsored by the National Institutes of Health [3]. 

Fatigue is the most common and disabling symptom among women successfully treated for breast cancer [4, 5]. Fatigue is elevated during cancer treatment [6] and persists beyond treatment completion in a substantial minority of women, with approximately 30% of breast cancer survivors reporting moderate to severe symptoms of fatigue one or more years post-treatment [7–9]. Cancer-related fatigue has been described by patients as a “different type of fatigue” as it is pervasive, debilitating, and not relieved by rest, and involves physical, mental, and emotional exhaustion [10, 11]. Fatigue has a negative impact on work, social relationships, and daily activities and causes significant impairment in overall quality of life among breast cancer survivors [9, 12, 13]. In longitudinal assessments of breast cancer survivors, we have found considerable stability in fatigue levels over time. For example, fatigue scores at 1–5 years post-diagnosis were highly correlated with fatigue scores at 5–10 years post-diagnosis in a longitudinal study of breast cancer survivors [14]. These findings suggest that persistent fatigue may not remediate without targeted treatment. 

Despite the prevalence of cancer-related fatigue, few evidence-based interventions are currently available to manage this symptom [15]. There is growing evidence that behavioral interventions may be effective in reducing fatigue among cancer patients undergoing treatment. For example, exercise interventions have shown positive effects on fatigue during treatment [16], as have psychosocial interventions that include stress management and relaxation techniques [17–19]. However, only a few behavioral intervention studies have been conducted with cancer survivors [20], and only one randomized trial specifically targeted survivors with persistent fatigue [21]. Cancer survivors with persistent fatigue may be unwilling or unable to participate in standard exercise interventions, such as walking or cycling; indeed, recent evidence suggests that fatigue is one of the primary barriers to participation in exercise programs among cancer survivors [22, 23]. 

Yoga is a promising alternative treatment for cancer survivors with persistent fatigue [24]. Yoga involves physical postures (*asanas*) that develop strength, flexibility, and relaxation and also promotes present awareness through the focus on body and breath in each pose [25]. A growing number of predominantly uncontrolled yoga intervention trials have been conducted among patients with a variety of medical conditions and have shown preliminary evidence of beneficial effects on physical and behavioral outcomes, including fatigue [26–28]. For example, a recent study found that patients with multiple sclerosis randomized to an Iyengar yoga intervention reported significant improvements in fatigue relative to a wait-list control [29]. Yoga interventions have also demonstrated positive effects on behavioral symptoms related to fatigue, including depressed mood [30–32], pain [33–35], and physical function [36]. 

A growing number of yoga interventions have been conducted with cancer patients. An early study conducted by Cohen and colleagues found that 7 weekly sessions of Tibetan yoga for patients with Stage I–IV lymphoma who were receiving or had recently completed chemotherapy led to significant improvements in sleep quality relative to a wait-list control, though no effects on fatigue or depression were observed [37]. This intervention included controlled breathing and visualization and simple yoga postures. Studies conducted with breast cancer patients and survivors, including several recent randomized trials, have also shown beneficial effects on quality of life, mood, and fatigue [38–47]. However, none of these studies specifically targeted patients with fatigue nor focused on fatigue as a primary outcome. This is significant given that fatigue was associated with lower adherence in a recent trial of yoga for breast cancer patients [47]. Moreover, all but one published trial [46] has included patients who were actively undergoing treatment. Thus, the feasibility and efficacy of yoga interventions for fatigued survivors have not been determined. 

The purpose of this pilot study was to evaluate the acceptability of an Iyengar yoga intervention for fatigued breast cancer survivors and to explore effects on fatigue and related outcomes. Iyengar yoga is a traditional form of Hatha yoga based on the teachings of B.K.S. Iyengar, a master practitioner and teacher [25, 48]. The Iyengar yoga system focuses on the therapeutic benefits of specific postures (*asanas*) and breathing techniques (*pranayama*) to address specific medical problems and conditions. Iyengar-based yoga interventions have been used in randomized trials for multiple sclerosis [29], carpal tunnel syndrome [33], osteoarthritis [34], lower back pain [35], and depression [31, 32]. For each condition, a prescribed series of poses formulated according to Mr. Iyengar's teachings was used, with beneficial effects seen on a variety of outcomes. This study used Iyengar principles to evaluate a series of postures and breathing techniques intended to address symptoms of fatigue in breast cancer survivors. We focused on Iyengar yoga because of its therapeutic orientation and its demonstrated efficacy in previous trials with medical patients. In addition, because there is a prescribed series of poses, Iyengar yoga is well-suited for empirical evaluation and dissemination.

## 2. Methods

### 2.1. Participants

 Breast cancer survivors with persistent fatigue were recruited through newspapers advertisements and flyers distributed in local oncology clinics. Fatigue was assessed with the vitality subscale of the SF-36, a reliable and valid 4-item scale that assesses how much of the time respondents feel “worn out”, “tired”, have “a lot of energy”, and feel “full of pep” over the past 4 weeks [49]. Scores on this scale range from 0–100, with scores below 50 indicating disability or limitations related to fatigue. Women who scored at or below 50 on this scale at both a phone and an in-person screen (conducted an average of 4 months apart) were eligible for study participation. In previous research, we have shown that breast cancer survivors scoring below 50 on the SF-36 show alterations in psychosocial, immune, and neuroendocrine function, supporting the validity of this cut-point [9, 50–54]. Other eligibility criteria included (1) 45–65 years old, (2) originally diagnosed with Stage 0–II breast cancer, (3) completed local and/or adjuvant cancer therapy (with the exception of hormonal therapy), (4) no breast cancer recurrence, (5) no chronic medical conditions or physical contraindications to the specific yoga sequences being taught, (e.g., immune-related disease, diabetes, neurological disease, uncontrolled depression, uncontrolled high blood pressure, BMI > 30, active respiratory problems or asthma, and moderate to severe neck, back, knee, or shoulder problems) (6) no current yoga practice (i.e., had not taken yoga classes regularly in past year), (7) nonsmoker, and (8) able to attend the scheduled classes. These criteria were designed to control for potential confounds that might influence fatigue levels and to ensure participant safety. 

A total of 62 women were screened by phone, 14 of whom met initial criteria and completed an in-person screen at UCLA. From this group, 12 were determined to be eligible for study participation and enrolled in the trial. Fifty women were not eligible: 8 scored above 50 on the SF-36 vitality subscale, 6 were older than 65 years old, 4 were actively receiving treatment for their breast cancer, 2 had breast cancer recurrences, 17 had chronic medical conditions and/or injuries (including 9 with a BMI > 30), 1 was engaged in a regular yoga program, 1 was a regular smoker, and 11 could not commit to the class schedule. One woman withdrew from the trial after several sessions because of a pre-existing knee condition and family-related demands. Thus, analyses are based on the 11 women who completed the full course of treatment. Demographic and medical characteristics of study participants are shown in Table 1. The mean score on the SF-36 vitality scale across the two screening assessments was 24.3, indicating severe fatigue. The UCLA Institutional Review Board approved the study procedures, and written informed consent was obtained from all participants.

### 2.2. Procedures

The yoga intervention was conducted in two groups. Participants in each group were assessed before and after the 12-week intervention and at a 3-month post-intervention follow-up. Pre- and post-assessments were conducted within 1-2 weeks of the intervention, and were not conducted on the same day as the yoga classes. Self-report measures were completed at each assessment point, and timed performance tasks were conducted at pre- and post-intervention. Women also completed weekly diaries assessing fatigue and other symptoms throughout the intervention. All 11 participants completed the pre- and post-intervention assessments; one woman withdrew before the 3-month post-intervention follow-up because of a breast cancer recurrence. Recruitment materials and the study consent form indicated that the purpose of the trial was to determine whether yoga classes could improve energy, mood, and physical function in breast cancer survivors.

### 2.3. Intervention

Iyengar yoga classes were conducted for 90 minutes twice a week for 12 weeks. This 12-week time frame was designed to accommodate all of the prescribed poses and to allow sufficient time for beneficial effects on energy to emerge. A certified Junior Intermediate Iyengar Yoga teacher taught the classes under the guidance of an Advanced Iyengar Yoga teacher at her home studio. The teacher was not blind to the study design or hypotheses. 

Iyengar yoga prescribes specific poses for individuals with specific medical problems and conditions; this trial emphasized postures believed to be effective for reducing fatigue among women with a history of breast cancer. Key postures from the trial are listed in Table 2 and include passive inversions (i.e., upside-down postures with support) and passive backbends (i.e., chest opening postures with support). In passive postures, the shape of the posture is supported by props (e.g., blocks, bolsters, blankets, wall ropes, and belts), rather than held by the strength of the body, so that participants can perform and maintain the postures without stress and tension. The postures were introduced systematically, progressing from mild to more challenging over the course of treatment. Most poses were held from 5 to 10 minutes. Breathing techniques were taught in a supine position with the chest supported by a bolster and the head resting on a blanket. This position is believed to provide an optimal opening of the chest and lungs for breathing without strain. 

Participants were not given “homework” or specifically instructed to practice postures outside of class; this was done to reduce participant burden. However, participants were not discouraged from home practice, and 7 women noted that they were practicing outside of class. 

### 2.4. Measures

The primary outcome of interest was subjective fatigue, which was assessed using the 14-item *Fatigue Symptom Inventory (FSI)* [55, 56]. The FSI assesses fatigue intensity, duration, and interference with daily functioning. This scale was developed for use with breast cancer patients and has been established as a reliable and valid measure of fatigue in cancer populations and healthy controls. The FSI is strongly correlated with other fatigue measures, including the fatigue scale of the POMS and the SF-36 vitality scale, demonstrating its convergent validity. We focused on items assessing fatigue severity and duration, including average fatigue in last week, most fatigue in last week, and number of days fatigued in last week. 

Secondary outcomes included depressed mood, sleep disturbance, pain, health-related quality of life, and measures of physical function. The *Beck Depression Inventory-II (BDI-II) *was used as a self-report measure of depressive symptomatology [57]. This 21-item scale has excellent reliability and validity and was associated with fatigue in our previous research with breast cancer survivors [50]. The *Pittsburgh Sleep Quality Index (PSQI)* was used to assess subjective sleep quality [58]. This 19-item questionnaire has been demonstrated to have high internal consistency, test-retest reliability, and diagnostic validity. The *Breast Cancer Prevention Trial (BCPT) Symptom Scale *was used to assess general musculoskeletal pain [59]. The *SF-36* was used to assess health-related quality of life, focusing on general health perceptions [49]. Participants also completed weekly diaries and post-intervention interviews to evaluate their perceptions of benefit and intervention efficacy. 

Timed performance tasks were used to provide an objective measure of physical function and were completed at pre- and post-intervention. These included the *8-foot Walk Test*, a commonly used measure of functional ability that is moderately correlated with oxygen consumption [60]. Participants were asked to walk as fast as they could for 8 feet and the total time was measured. *Timed Chair Stands* were used to assess lower extremity strength and endurance [61]. Participants were asked to stand up and sit down 5 times in an armless, straightback chair, and their performance timed. For both tasks, performance times were converted into scores following standard procedures, with higher scores indicating faster performance. Both tasks are validated measures of physical function that can be used with middle- to older-age individuals. 

## 3. Results

One of the main goals of this pilot study was to evaluate the acceptability of an Iyengar yoga intervention for breast cancer survivors with persistent fatigue. Of the 12 women enrolled in the trial, 11 completed the full 12-week course of treatment, supporting the acceptability of the intervention. The average number of classes attended was 22.4 (93% of classes offered; range = 19–24 classes attended); reasons for missing class included illness, vacation, and travel. At interviews conducted after treatment, all participants reported that the intervention was beneficial, that they planned to continue with yoga, and would recommend the intervention to other fatigued cancer survivors. At the 3-month post-intervention follow-up, all but one participant reported that they were continuing to take yoga classes, and all but one (the one who had not continued with yoga) reported that they had experienced enduring benefits from the intervention. 

### 3.1. Improvement in Fatigue after Yoga Intervention

The primary outcome of interest in this trial was subjective fatigue. Paired samples *t*-tests showed significant improvement on the Fatigue Symptom Inventory from pre- to post-intervention, including decreases in average fatigue (mean change = 3.5, *t*(10) = 5.7, *P* < .001), most fatigue (mean change = 3.8; *t*(10) = 4.7, *P* = .001), and number of days fatigued in last week (mean change = 2.4, *t*(10) = 2.9, *P* < .05), as shown in Table 3. We also saw improvements on the vitality subscale of the SF-36, as indicated by an increase in vitality scores (mean change = 22.7, *t*(10) = −4.2, *P* = .002; note that higher scores on this scale indicate more energy/less fatigue). Further, there was evidence that these changes persisted over the 3-month post-intervention follow-up, as changes on each measure from baseline to 3-month post-intervention follow-up were significant (average fatigue: mean change = 1.9, *t*(9) = 3.9, *P* = .003; most fatigue: mean change = 2.2, *t*(9) = 3.2, *P* = .01; days fatigued: mean change = 2.5, *t*(9) = 3.6, *P* = .005; SF-36 vitality: mean change = 18.5, *t*(9) = −4.2, *P* = .002) but changes from post-intervention to 3-month post-intervention follow-up were not (all *P*s > .05). Analyses conducted using Wilcoxon signed-ranked tests yielded the same results.

### 3.2. Improvement in Secondary Outcomes after Yoga Intervention

There was a significant decrease in depressive symptoms on the BDI-II from pre- to post-intervention (mean change = 7.9, *t*(10) = 3.3, *P* = .008) that persisted at the 3-month post-intervention follow-up (mean change = 6.7, *t*(9) = 3.4, *P* = .007). There was also significant improvement on the general health subscale of the SF-36 (mean change = 14.5, *t*(10) = 3.5, *P* = .005) that persisted at the 3-month post-intervention follow-up (mean change = 14.3, *t*(9) = 5.1, *P* = .001). Changes from post-intervention to 3-month post-intervention follow-up were not significant for depression or general health. 

There was a trend towards a decrease in pain on the BCPT symptom checklist from baseline to post-intervention (*P* = .07), but no change from baseline to 3-month follow-up was observed. There was no significant change in sleep quality on the PSQI at either assessment point (*P*s > .3). Analyses conducted using Wilcoxon signed-ranked tests yielded the same results. Exploratory analyses conducted on the remaining six SF-36 subscales (physical function, role function-physical, mental health, role function-emotional, pain, and social function) evidenced significant improvements in role function-physical and social function (both *P*s < .01); improvements in social function persisted at the 3-month follow-up (*P* < .01). Although positive changes were observed in all SF-36 subscales, none of the other changes reached statistical significance. 

**Figure 1:**
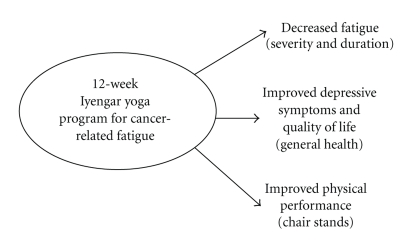
Demonstrates positive outcomes of a 12-week Iyengar Yoga program for cancer-related fatigue in breast cancer survivors.

On the performance measures, there was a significant improvement in chair stands from baseline to post-intervention (i.e., participants were able to perform significantly more chair stands after the intervention; *t*(10) = −3.6, *P* = .005), although no changes in the 8-foot walk test were observed. Of note, average scores on this test approached the scale maximum of 4 at each assessment, suggesting that there may have been a ceiling effect.  Figure 1 summarizes improvements in both primary and secondary outcomes of interest.

### 3.3. Representative Comments from Study Participants

Participants' comments in their weekly diaries provided additional insight into their intervention experience. One participant began to notice a change in her energy at week 3: “The physical and mental changes are subtle, but I feel like I'm thinking more clearly, have less brain fog, have more energy, fatigue later rather than sooner, feel better in general, feel stronger." At week 10, she wrote: “I'm really aware this week how much more energy I have to do more things in a week. I wouldn't describe it as “energetic,” I'd just say that I have reserves.” Another participant summed up her experience at week 12 as follows: “(The) effects of yoga have been small each week but the cumulative effect has been noticeable. I know that it is ameliorating the fatigue…. There's been enough progress, subtle and incremental, to believe I can get much better.”

## 4. Conclusions

Results from this small, uncontrolled pilot study support the feasibility and acceptability of a tailored Iyengar yoga intervention for breast cancer survivors with persistent fatigue. Although the women had minimal prior yoga experience, they were enthusiastic about the classes and adherence was excellent; indeed, almost all participants reported that they continued taking yoga classes after the 12-week intervention was completed, and many had purchased props so that they could perform postures at home. Moreover, they were willing to attempt all of the postures and reported no adverse effects. We speculate that the careful selection of postures that would be appropriate for this patient population, the sequencing of postures from mild to more challenging over the course of the intervention, the in-depth screening of study participants and careful supervision throughout the trial, and instruction by an experienced teacher all contributed to treatment feasibility and acceptability. 

Results also provide preliminary support for the efficacy of this intervention in reducing symptoms of fatigue. Scores on each of the fatigue measures showed a significant improvement from pre- to post-intervention; for example, scores on the “average fatigue” item of the FSI fell from 6.3 to below 3, the cut-off for clinically significant fatigue [62], and scores on the SF-36 vitality scale increased by 22.7 points, a clinically significant change [63]. Further, improvements were maintained at the 3-month post-intervention follow-up. This can be contrasted with effects seen in research on exercise interventions for cancer survivors, which have typically shown weaker effects on subjective fatigue [64]. These findings are particularly encouraging given that study participants had experienced fatigue for several years before trial onset (mean number of years since diagnosis = 4.6; range = 10 months–15 years). Of note, participants continued to score below population norms on the SF-36 vitality scale (60.6 for women aged 45–54) at both post-treatment assessments, suggesting that a longer intervention may be required to further increase energy levels among women who have suffered from fatigue for many years. 

Intervention effects extended beyond subjective symptoms of fatigue to objective measures of physical performance, specifically chair stands. These findings suggest that the intervention may have beneficial effects on strength in addition to subjective symptoms. No changes were seen in the 8-foot walk test, although this may have been due to the restricted range of scores on this measure. Improvements were also seen in depressive symptoms and perceptions of general health. At the baseline assessment, average scores on the Beck Depression Inventory-II fell in the “mildly depressed” range (14–19), whereas average scores at follow-up fell in the “minimally depressed” range (0–13). On the SF-36 general health scale, average scores fell below the population norm of 70.5 at both assessments. Of note, multiple statistical tests were conducted to evaluate these secondary outcomes, increasing the risk of Type I errors. 

Although these results are promising, the study has several key limitations that render the findings preliminary. The first is the small sample size; only 11 women participated in the intervention. These women are demographically and medically similar to a large cohort of fatigued breast cancer survivors we have previously characterized [9], suggesting that they may be roughly representative of this population of women. Clearly, though, a larger sample is required to demonstrate intervention efficacy. It is also important to note that the women in this trial were carefully screened for medical and physical problems that might limit their ability to safely perform the required poses; approximately 25% of the women screened for study participation were excluded because of such comorbidities. Thus, this particular practice may be best suited for a relatively high functioning population. The second major limitation of this trial is the lack of a control group. It is possible that improvements in fatigue might have occurred in the absence of any intervention, although our previous research indicates that fatigue scores are quite consistent over time in women with persistent post-treatment fatigue [14]. In addition, it is possible that nonspecific aspects of the intervention (e.g., social support) may have influenced fatigue scores. Expectations about treatment efficacy may also have influenced outcomes; participants were told that the intervention was designed to increase energy, and the yoga instructor reinforced this perspective. We are currently conducting a randomized controlled trial of this Iyengar yoga intervention which will provide a stronger test of its efficacy and will also enable us to test potential mechanisms for treatment effects. 

## Figures and Tables

**Table 1:** Demographic and medical characteristics of study participants.

Characteristic	*N* = 11
Age	
Mean	53.8
Range	46–65
Ethnicity, no.	
White	9
Nonwhite	2
Married or in significant relationship, no.	
Yes	10
No	1
Education level, no.	
High school graduate	1
College graduate	4
Completed Master's or Doctoral degree	6
Employment status, no.	
Employed full or part time	9
Retired/unemployed	2
Income, no.	
<$45,000	1
$45,000–$75,000	3
>$75,000	7
Type of treatment, no.	
Surgery only	3
Surgery + radiation	4
Surgery + radiation + chemotherapy	4
Years since diagnosis	
Mean	4.6
Range	10 mo–15 yrs

**Table 2:** Key yoga postures for breast cancer survivors with fatigue. The following poses appear out of sequence and are not in the order in which they were taught.

Sanskrit name	Description
Supta Baddhakonasana	Reclining bound angle posture
Supta Svatstikasana	Reclining cross legged posture
Setubandha Sarvangasana on bolsters	Bridge pose on cross bolsters
Setubandha Sarvangasana on bench	Bridge pose on bench with Viparita Karani box
Purvottanasana	Extension of the front body supported on two chairs
Viparita Dandasana on two chairs	Backbend over two chairs with thoracic support
Salamba Sarvangasana on a chair	Shoulder stand on a chair
Salamba Sirsasana on ropes	Hanging rope headstand
Supta Konasana with two chairs	Variation of Halasana, plow pose, with legs spread apart and feet supported on two chairs
Viparita Karani	Inverted lake pose
Bharadvajasana on chair	Seated chair twist
Adhomukha Svanasana on ropes with chair	Downward facing dog posture on ropes with chair
Urdhva Mukha Svanasana with chair	Upward facing dog posture with chair support
Tadasana Urdhva Hastasana	Mountain posture with arms stretched up
Tadasana Urdhva Baddha Hastasana	Mountain posture with bound hands
Savasana	Corpse posture with bolster support under chest

**Table 3:** Changes in fatigue and other outcomes following pilot yoga intervention.

	Baseline	Post-treatment	3-month follow-up
Primary outcomes: (range)	Mean (SD)	Mean (SD)	Mean (SD)

FSI average fatigue (0–10)	6.3 (1.1)	2.7 (1.6)**	4.4 (1.8)**
FSI #days fatigued (0–7)	7 (0)	4.6 (2.7)*	4.5 (2.2)**
FSI most fatigue (0–10)	7.8 (1.3)	4.0 (2.3)**	5.8 (2.3)**
SF-36 vitality scale (0–100)	27.3 (18.4)	50 (22.2)**	47.5 (18.4)**

Secondary outcomes: (range)	Mean (SD)	Mean (SD)	Mean (SD)

Depressive symptoms (BDI-II) (0–63)	15.4 (8.0)	7.5 (6.2)**	7.3 (5.6)**
SF-36 general health (0–100)	50.5 (22.1)	65.0 (22.1)**	67.3 (23.9)**
General pain (BCPT) (0–4)	1.5 (1.4)	0.8 (0.6)	1.3 (0.9)
Sleep quality (PSQI) (0–21)	7.1 (4.1)	6.4 (3.6)	6.0 (3.7)
Chair stand score (0–4)	2.6 (0.9)	3.5 (0.5)	n/a
8-foot walk test (0–4)	3.4 (0.6)	3.4 (0.6)	n/a

Paired-samples *t*-tests were used to evaluate differences from baseline to post-treatment and from baseline to 3-month follow-up.

*P < .05; **P < .01.
